# The Influence of the Ceramic Nanoparticles on the Thermoplastic Polymers Matrix: Their Structural, Optical, and Conductive Properties

**DOI:** 10.3390/polym13162773

**Published:** 2021-08-18

**Authors:** Ion Smaranda, Andreea Nila, Paul Ganea, Monica Daescu, Irina Zgura, Romeo C. Ciobanu, Alexandru Trandabat, Mihaela Baibarac

**Affiliations:** 1 Laboratory Optical Processes in Nanostructured Materials, National Institute of Materials Physics, Atomistilor Street 405A, R077125 Bucharest, Romania; ion.smaranda@infim.ro (I.S.); andreea.nila@infim.ro (A.N.); paul.ganea@infim.ro (P.G.); monica.daescu@infim.ro (M.D.); irina.zgura@infim.ro (I.Z.); 2 Department of Electrical Measurements and Materials, Faculty of Electrical Engineering, Technical University Gh. Asachi Iasi, Boulevard Profesor Dimitrie Mangeron 67, R070050 Iasi, Romania; rciobanu@yahoo.com (R.C.C.); ftranda@yahoo.com (A.T.)

**Keywords:** thermoplastic polymers, ceramic nanoparticles, Raman scattering, IR spectroscopy, photoluminescence, dielectric spectroscopy

## Abstract

This paper prepared composites under the free membranes form that are based on thermoplastic polymers of the type of polyurethane (TPU) and polyolefin (TPO), which are blended in the weight ratio of 2:1, and ceramic nanoparticles (CNs) such as BaSrTiO_3_ and SrTiO_3_. The structural, optical, and conductive properties of these new composite materials are reported. The X-ray diffraction studies highlight a cubic crystalline structure of these CNs. The main variations in the vibrational properties of the TPU:TPO blend induced by CNs consist of the following: (i) the increase in the intensity of the Raman line of 1616 cm^−1^; (ii) the down-shift of the IR band from 800 to 791 cm^−1^; (iii) the change of the ratio between the absorbance of IR bands localized in the spectral range 950–1200 cm^−1^; and (iv) the decrease in the absorbance of the IR band from 1221 cm^−1^. All these variations were correlated with a preferential adsorption of thermoplastic polymers on the CNs surface. A photoluminescence (PL) quenching process of thermoplastic polymers is demonstrated to occur in the presence of CNs. The anisotropic PL measurements have highlighted a change in the angle of the binding of the TPU:TPO blend, which varies from 23.7° to ≈49.3° and ≈53.4°, when the concentration of BaSrTiO_3_ and SrTiO_3_ CNs, respectively, is changed from 0 to 25 wt. %. Using dielectric spectroscopy, two mechanisms are invoked to take place in the case of the composites based on TPU:TPO blends and CNs, i.e., one regarding the type of the electrical conduction and another specifying the dielectric–dipolar relaxation processes.

## 1. Introduction

Composite materials based on ceramic nanoparticles (CNs) and macromolecular compounds have shown improved features of processability, dielectric and mechanical properties responsible for high motivating economic impact, and high-level applications [1,2]. Even though BaTiO_3_ is the most desirable ceramic filler because of its friendly environment character and high dielectric constant, depending on the synthesis procedure and particle size [3], another promising perovskite material is SrTiO_3_, which also exhibits a strong dielectric constant of about 300 and a low dielectric loss of 0.02 [4]. Furthermore, an exciting new material that brings together the features of both BaTiO_3_ and SrTiO_3_ is BaSrTiO_3_. This is also a perovskite-type material with a high insulation resistance and advantageous dielectric properties [5]. As a result of their high dielectric permittivity and low dielectric loss, BaTiO_3_, its SrTiO_3_ counterpart, and their BaSrTiO_3_ compositions are the most common dielectric ceramics used in communication [6], micro- and nano-electronics [7], or new generation photonics [8]. However, for 3D printing technology and other flexible device applications, the poor flexibility of BaTiO_3_, SrTiO_3_, and BaSrTiO_3_ limits their applications at a large scale. The progress of a filler or nanoparticles-incorporated polymer matrix is a way to overcome these limitations and develop more useful related composite structures exhibiting combined improved dielectric and mechanical properties [2], which extend the spectrum of applications regarding 3D printing. On the other side, in order to expand the physicochemical properties of polymer-based 3D printable materials used in various areas of complex geometry manufacturing, the incorporation of nanoparticles or filler in the polymeric matrix has been widely investigated (e.g., [1]). As a result of their easy processability, flexibility, and high mechanical properties, polymers were the most prevailing matrix used for incorporating other nanoparticles [2]. From this category, thermoplastic elastomers represent promising candidates due to their capability to tolerate a high degree of deformation, and thus, they represent a class of materials that can be easily processed by polymer-based 3D printing technology. In this direction, thermoplastic polymers of the type of poly(vinylidene fluoride) (PVDF), acrylonitrile–butadiene–styrene (ABS), polycarbonate (PC), or polyphenylsulfone (PPSU) are frequently observed in the literature [1,9]. However, their low functional behavior causes a decrease in shear transfer interaction with the matrix [1]. This limitation can be overcome by using the thermoplastic polyurethane (TPU), which is an elastomeric material that possesses both soft and hard urethane segments in its chemical structure composition. For instance, in addition to its great flexibility, TPU has a low modulus and a high elongation, reaching up to 600% in the case of fiber-reinforced composites [10]. Moreover, the glassy and rubbery features of TPU are suitable for fused deposition processing [10]. Nevertheless, to increase the processability and thermal stability of TPU, thermoplastic polyolefin (TPO) elastomers have been chosen to make blends of TPU:TPO [11] as an efficient method of achieving the demanding performances not provided by a homopolymer.

Regarding these ceramic-based thermoplastic elastomer composites, there are already several works that confirm their connected features given by both elastomers and the incorporated nanoparticles with high dielectric characteristics, which could be also applicable as active composites for 3D printing technology [9,12,13,14]. After our best knowledge, to date, no study has been reported on composites based on TPU:TPO blends and CNs of the type BaSrTiO_3_ or SrTiO_3_. In comparison with the our previous study reported on the composites based on the TPU:TPO blends and the BaTiO_3_ particles [12], in this work, new information will be reported about the following: (i) the adsorption of thermoplastic polymers onto the surface of CNs of the type BaSrTiO_3_ and SrTiO_3_, (ii) the role of CNs, BaSrTiO_3,_ and SrTiO_3,_ onto the photoluminescence process of the TPU:TPO blend, and (iii) the dielectric and conductive proprieties of the composites based on the TPU:TPO and CNs as well as their mechanisms that take place. In order to highlight this progress, herein, we report structural, optical, dielectric, and conductive properties of the TPU:TPO/CNs composites using X-ray diffraction (XRD), Raman scattering, Fourier-transform infrared (FTIR) absorption, photoluminescence, and dielectric spectroscopy as investigation methods. According to our previous work [12], a weight ratio of 2:1 for TPU:TPO blends was chosen in order to annihilate the effect of surrounding the adjacent segments of TPU in the case of a high concentration of TPO. The influence of the CNs concentration in the case of BaSrTiO_3_ and SrTiO_3_ on the structural, optical, dielectric, and conductive properties of the TPU:TPO 2:1 blend will be also reported. In this context, free membranes of composites based on the TPU:TPO 2:1 blend with various weight percentages concentrations of CNs, e.g., 6 wt %, 12 wt % and 25 wt %, will be prepared and used for optical and dielectric tailor-made responses of these proposed materials. The detailed and complex characterization will highlight the viability of these composites for the various applications as a guide to be further processed through 3D printing technologies.

## 2. Materials and Methods

CNs of the type BaSrTiO_3_ and SrTiO_3_ as well as dimethyl formamide (DMF) and ethanol were bought from Sigma-Aldrich, while the thermoplastic polymers, i.e., TPU and TPO, and the curing agent of TPO were purchased from Elastollan-BASF Chemical Company R10201. According to the specification sheets of BaSrTiO_3_ and SrTiO_3_, the two CNs have the size <100 nm.

The preparation of the composite membranes based on the TPU:TPO 2:1 blend and CNs was performed according to ref. [12]. Briefly, the TPU:TPO 2:1/CNs membranes were prepared as follows. (i) The solution of TPU in DMF (0.062 g/mL) was mixed ultrasonically with a solution of TPO in ethanol (0.05 g/mL) containing 0.1 g of polydimethylsiloxane, which has the role of hardener of TPO. (ii) Different amounts of CNs, i.e., 0.05, 0.1, and 0.23 g were added to each mixture of the TPU and TPO solutions, which after the dispersion under ultra-sonication were poured in Petri dishes and annealing treated at 100 °C, at a time of 2 h. (iii) Finally, the films of the TPU:TPO 2:1/CNs composites, having the CNs concentration equal to 6 wt %, 12 wt % and 25 wt %, were peeled and dried under vacuum until it was found that their mass remain constant. In the following, the composites based on TPU:TPO 2:1 and CNs having the inorganic nanoparticles concentration equal to 6 wt %, 12 wt % and 25 wt % are labeled in the case of: (i) BaSrTiO_3_, as A, B, and C samples, while for (ii) SrTiO_3_, as D, E, and F samples, respectively.

X-ray diffraction (XRD) patterns of BaSrTiO_3_, SrTiO_3_, and the A, B, C, D, E, and F samples were performed with a Bruker D8 Advance diffractometer in the Bragg–Brentano configuration (Bruker AXS, Karlsruhe, Germany)

Raman spectra of the BaSrTiO_3_ and the A, B, C, D, E, and F samples were recorded with a MultiRAM FT-Raman spectrometer, from Bruker (Bruker Optik GmbH, Ettlingen, Germany).

IR spectra of the A, B, C, D, E, and F samples were recorded in attenuated total reflection (ATR) geometry with a Vertex 80 FTIR spectrophotometer from Bruker (Billerica, Massachusetts, US).

Photoluminescence (PL) spectra of the BaSrTiO_3_, SrTiO_3_, and the A, B, C, D, E, and F composites were recorded in right angle geometry of the FL3-2-2-1 Fluorlog-3 spectrophotometer from Horiba Jobin Yvon (Palaiseau, France).

Concerning the dielectric properties of the A, B, C, D, E, and F composites, the equipment for determining the complex dielectric function consists of a high-resolution “Alpha-A Frequency Analyzer” from Novocontrol GmBH; it works in the frequency range from 30 µHz to 20 MHz. The equipment works in the impedance range from 0.01 to 1014 Ω. Relative accuracy for impedance or capacitance measurements is less than 3 × 10^−5^, while the absolute accuracy of the phase angle measurement is less than 2 × 10^−5^ degrees. The measuring system has a temperature control unit, QUATRO-POWER, which allows temperature measurement with an accuracy of 0.01 °C and stabilization with an accuracy of 0.1 °C. The variation of the dielectric properties with the temperature in the range 0–100 °C was studied. The temperature was modified with a step of 5 °C.

## 3. Results and Discussion

### 3.1. Structural Properties of SrTiO_3_ and BaSrTiO_3_ and Their Composites with the TPU:TPO

**Figure 1:**
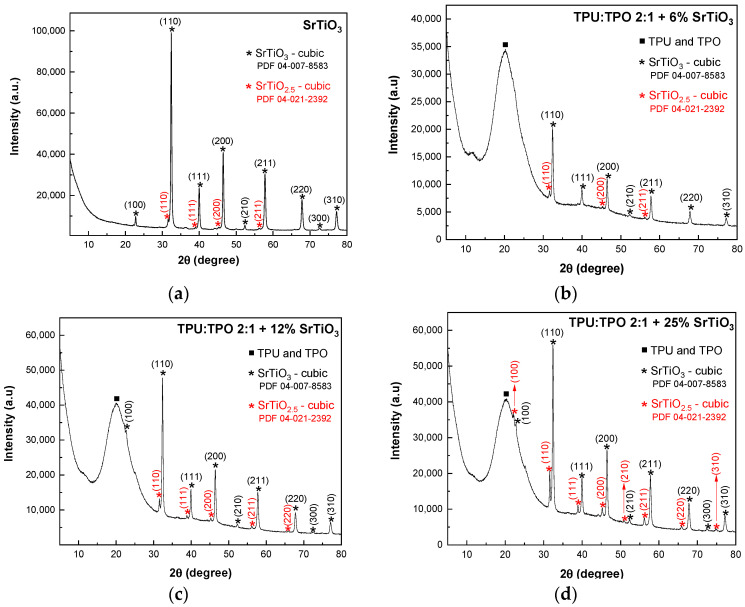
XRD patterns of SrTiO_3_ (**a**) and its composites containing blends of TPU:TPO 2:1 with varied concentrations of SrTiO_3_, i.e., 6 wt % (**b**), 12 wt % (**c**) and 25 wt % (**d**). The crystallographic planes of constituents are represented with black and red stars for SrTiO_3_ and SrTiO_2.5_, respectively, while the full squares define the amorphous phase of TPU:TPO blends.

**Figure 2:**
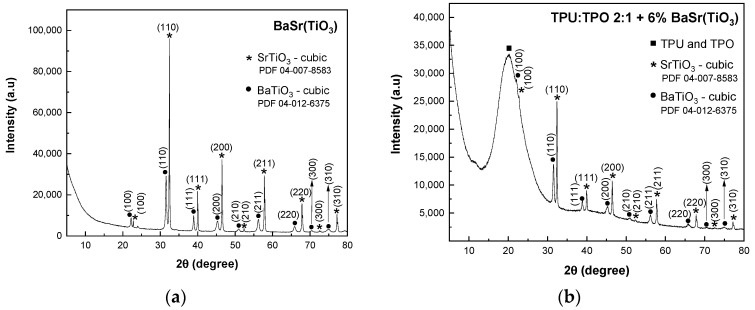

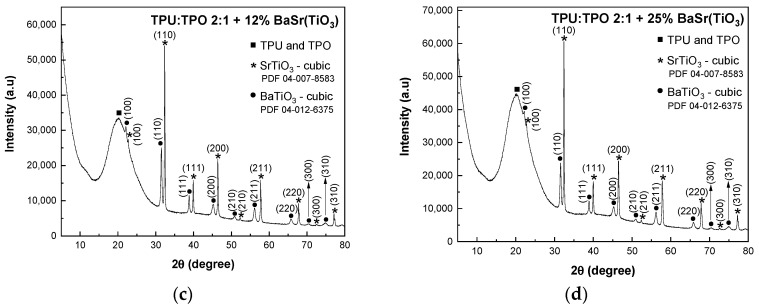
XRD patterns of BaSrTiO_3_ (**a**) and its composites containing the blends of TPU:TPO 2:1 with varied concentrations of BaSrTiO_3_, i.e., 6 wt % (**b**), 12 wt % (**c**), and 25 wt % (**d**). The crystallographic planes of constituents are represented with stars and cycle for SrTiO_3_ and BaTiO_3_, respectively, while the full squares define the amorphous phase of TPU:TPO blends.

The XRD patterns of SrTiO_3_ and BaSrTiO_3_ and their composites with the TPU:TPO 2:1 blends are presented in Figure 1 and Figure 2, indicating a high purity of the samples. The XRD pattern from Figure 1a shows a main cubic phase of SrTiO_3_ with the diffraction peaks attributed to the (100), (110), (111), (200), (210), (211), (220), (300), and (310) crystallographic planes, according to the PDF 04-007-8583 database. In addition to this phase, a secondary phase of SrTiO_2.5_ is revealed, and the peaks are associated with the (110), (111), (200), and (211) planes (PDF 04-021-2392 database). In composites-assisted blends of TPU:TPO 2:1 and SrTiO_3_ nanoparticles, the XRD patterns from Figure 1b–d show an amorphous phase located at a maximum 2θ of 20.2° attributed to the non-crystalline structure of TPU:TPO blends. The crystallites’ size was determined with the Scherrer equation, D = k × λ/(B cos θ), where D, k, λ, B, and θ correspond to the crystallite size, the shape factor, the X-ray wavelength, the half-maximum of the diffraction peak, and the Bragg angle. According to the results shown in Figure 1, the crystallite size varies with a slight decrease from 27.4 nm (SrTiO_3_ sample) to 24.6 nm (TPU:TPO 2:1 + 25%SrTiO_3_), 24.5 nm (TPU:TPO 2:1 + 12% SrTiO_3_), and 24.3 nm (TPU:TPO 2:1 + 6% SrTiO_3_).

In Figure 2a, the XRD pattern of BaSrTiO_3_ shows the crystallographic plane of SrTiO_3_ and the characteristic peaks of a cubic structure of BaTiO_3_ identified with the crystallographic planes of (100), (110), (111), (200), (210), (211), (220), (300) and (310) (PDF 04-12-6375 database).

In BaSrTiO_3_, the cubic phase of BaTiO_3_ overlaps the characteristic peaks of the SrTiO_2.5_ compound. Similar to the previous mixtures, in this case, the composites of BaSrTiO_3_ and TPU:TPO blends show in the XRD patterns (Figure 2b–d) the presence of the amorphous broad peak of TPU:TPO blends. Similar to the previous composites, the crystallite sizes have a decrease with decreasing the concentration of BaSrTiO_3_, varying from 47 nm (BaSrTiO_3_ sample) to 38.5 nm (TPU:TPO 2:1 + 25 wt % BaSrTiO_3_). 37.2 nm (TPU:TPO 2:1 + 12 wt % BaSrTiO_3_) and 35.2 nm (TPU:TPO 2:1 + 6 wt % BaSrTiO_3_). The decrease in the crystallites size must to be correlated with the ultra-sonication process of CNs carried out in the mixture of the thermoplastic polymers, TPU and TPO, when there is a dispersion of these nanoparticles in the macromolecular compounds mass, assisted by the polymers’ adsorption onto the crystallite surface, similar to that reported in the case of BaTiO_3_ [12], which will prevent their reconnection.

### 3.2. The Vibrational Properties of the Composites Based on TPU:TPO and the SrTiO_3_ and BaSrTiO_3_ CNs

**Figure 3:**
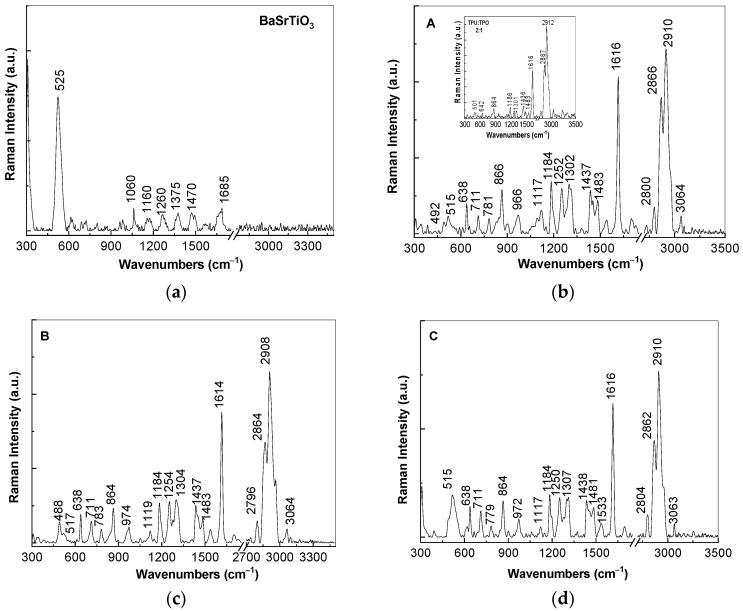
Raman spectra of BaSrTiO_3_ (**a**) and their composites with the TPU:TPO thermoplastic polymers, labeled as A, B, and C samples, having the CNs concentration equal to 6 wt % (**b**), 12 wt % (**c**), and 25 wt % (**d**). The insert of Figure (**b**) corresponds to the Raman spectrum of the TPU:TPO blend.

At the excitation wavelength of 1064 nm, Raman spectrum of (i) BaSrTiO_3_ is dominated of an intense Raman line with the maximum at 525 cm^−1^ (Figure 3a) assigned to the A_1_(TO) vibrational mode [15], while in the case of (ii) the TPU:TPO blend, an intense Raman complex band at 2867–2912 cm^−1^ accompanied by other lines of low intensity with peaks at 1616, 1436 and 1301 cm^−1^ is reported. The assignment of the Raman lines of the TPU:TPO blend is shown in Table 1.

**Figure 4:**
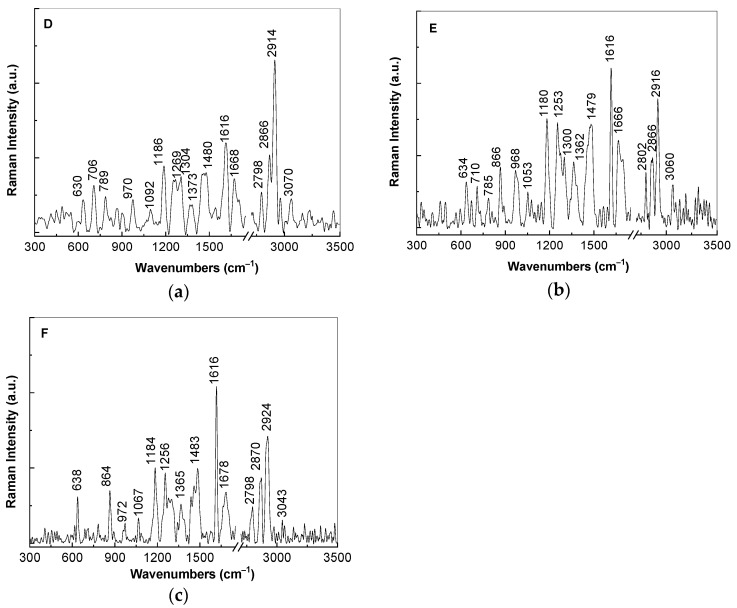
Raman spectra of the composites based on thermoplastic polymers TPU:TPO and SrTiO_3_, labeled as D, E, and F samples, when the CNs concentration is equal to 6 wt % (**a**), 12 wt % (**b**), and 25 wt % (**c**).

**Figure 5:**
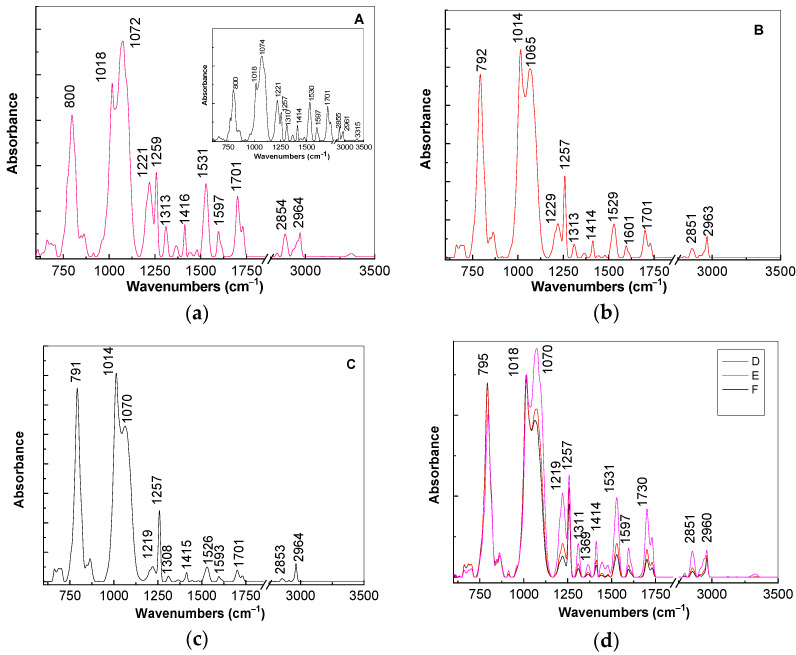
IR spectra of the composites based on thermoplastic polymers TPU:TPO 2:1 and BaSrTiO_3_ having the concentration of CNs equal to 6 wt % (**a**), 12 wt % (**b**), and 25 wt % (**c**). The insert in Figure (**a**) shows IR spectrum of the TPU:TPO blend. In Figure (**d**) are shown IR spectra of the composites based on the TPU:TPO blend and SrTiO_3_ having the concentration of CNs equal to 6 wt % (magenta curve), 12 wt % (red curve), and 25 wt % (black curve).

**Figure 6:**
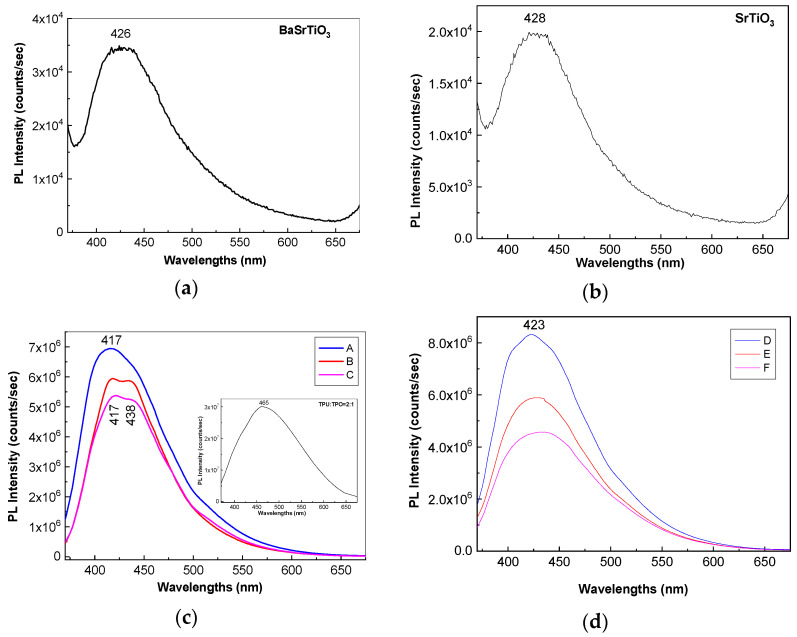
PL spectra of BaSrTiO_3_ (**a**), SrTiO_3_ (**b**), and the composites based on the TPU:TPO thermoplastic polymers and CNs of the type BaSrTiO_3_ (**c**) and SrTiO_3_ (**d**). In Figure (**c**,**d**), the PL spectra of: (i) the A and D samples are shown with blue lines, (ii) the B and E samples are shown with the red curve; and (iii) the C and F samples are shown with the magenta curve. The insert in Figure (**c**) shows the PL spectrum of the TPU:TPO blend.

**Figure 7:**
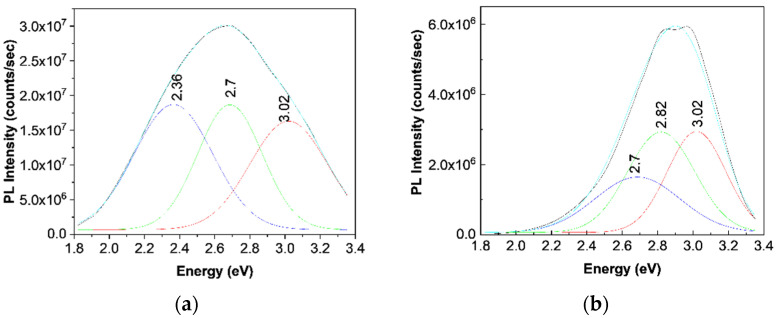
The deconvolution of PL spectra of the TPU:TPO blend (**a**) and the B sample (**b**).

**Figure 8:**
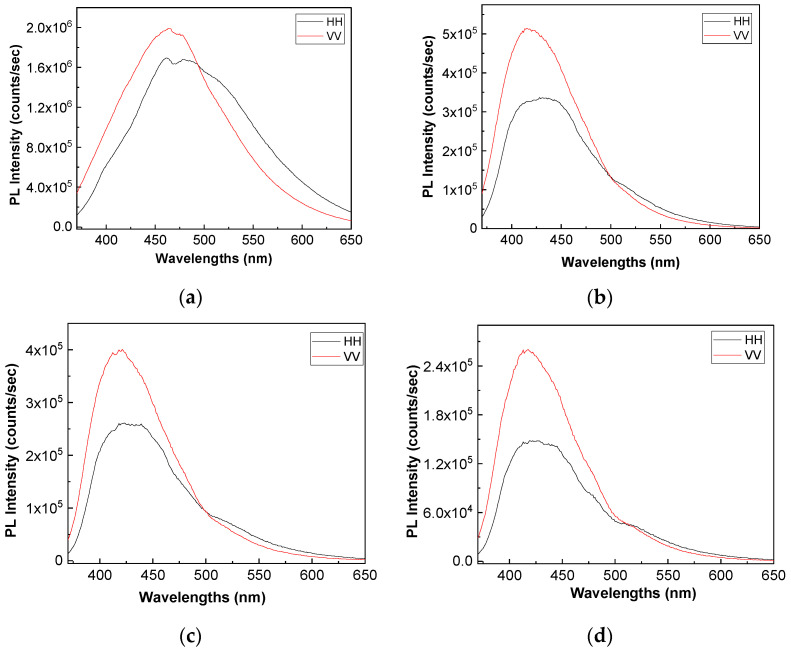
PL spectra in polarized light of the TPU:TPO thermoplastic polymers (**a**) and their composites with BaSrTiO_3_, when the CNs concentration is equal to 6 wt % (A sample, **b**), 12 wt % (B sample, **c**) and 25 wt % (C sample, **d**). All PL spectra were recorded at the excitation wavelength of 350 nm.

**Figure 9:**
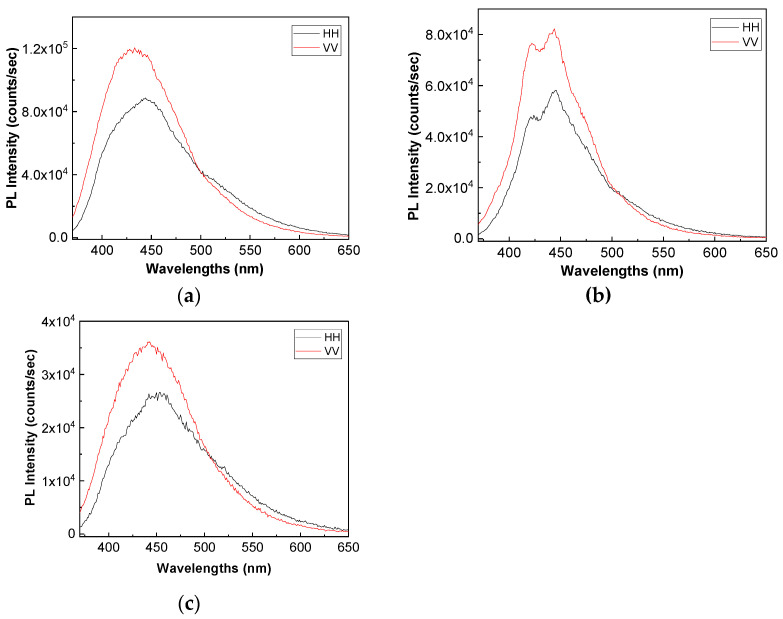
PL spectra in polarized light of the composites based on the TPU:TPO thermoplastic polymers and SrTiO_3_, when the CNs concentration is equal to 6 wt % (D sample, **a**), 12 wt % (E sample, **b**) and 25 wt % (F sample, **c**).

**Figure 10:**
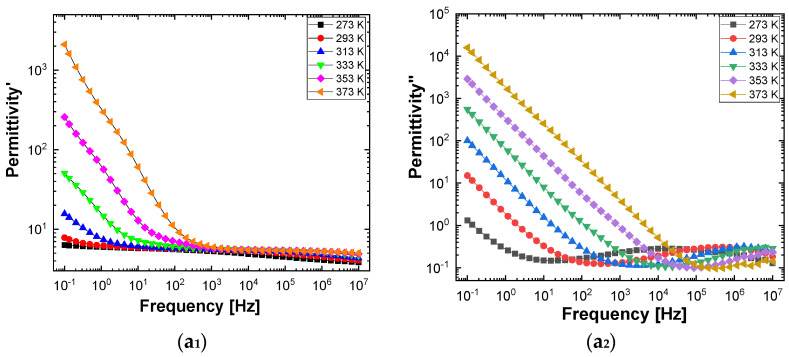

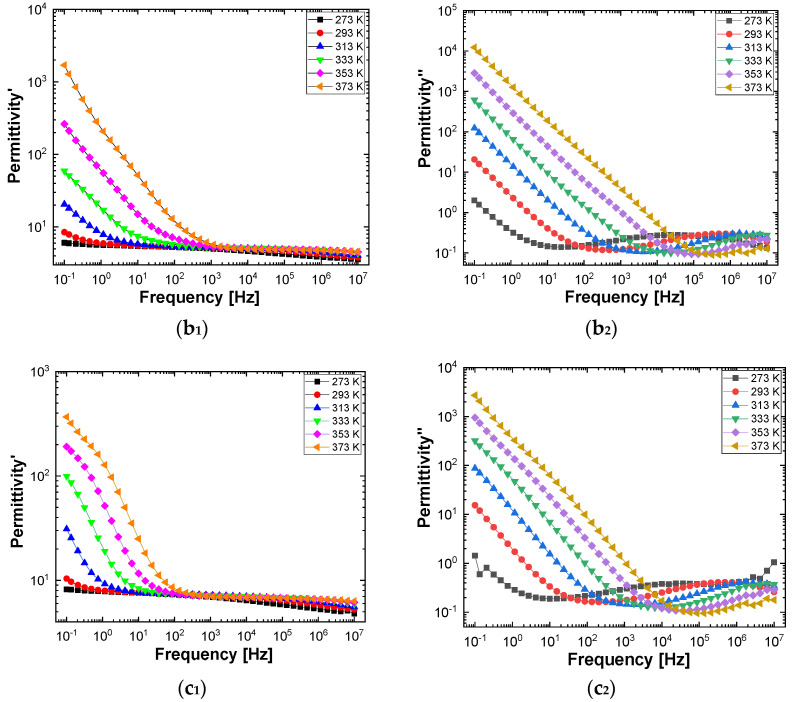
The spectra of permittivity (**1**) and dielectric loss (**2**) in the case of the samples labeled as A (**a_1_**,**a_2_**), B (**b_1_**,**b_2_**), and C (**c_1_**,**c_2_**), recorded at the temperature of 273 °K (black symbol), 293 °K (red symbol), 323 °K (blue symbol), 333 °K (green symbol), 353 °K (magenta symbol), and 373 °K (orange symbol).

**Figure 11:**
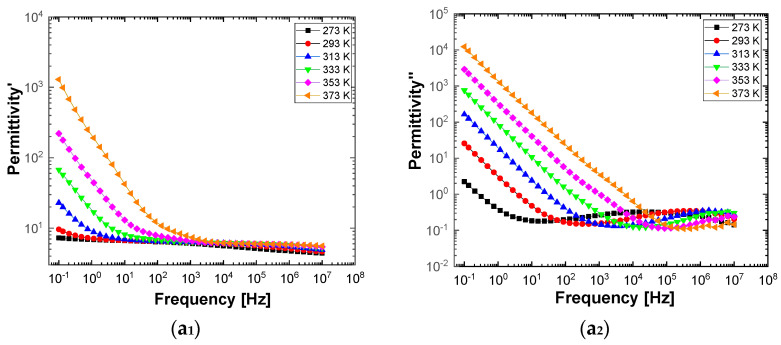

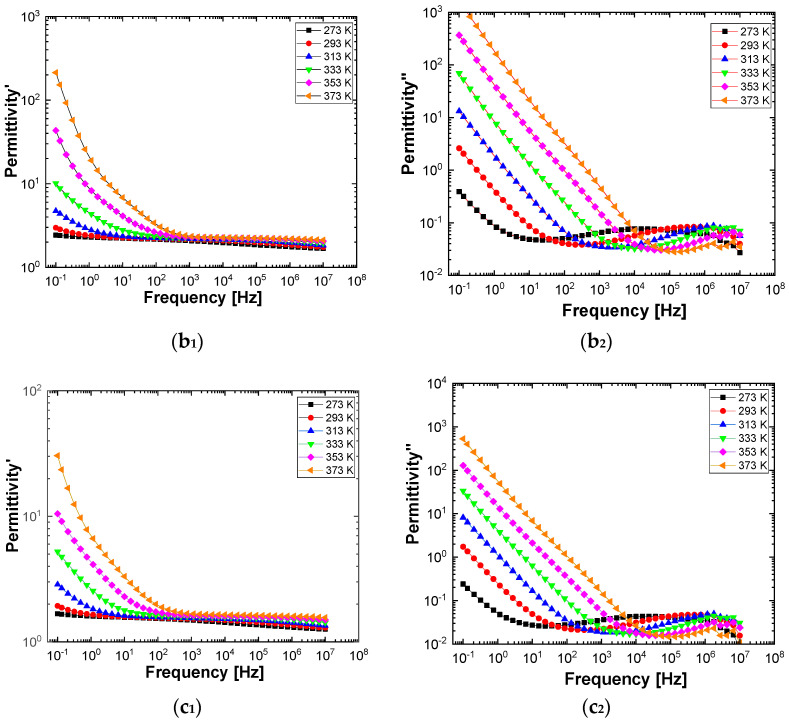
The spectra of permittivity (**1**) and dielectric loss (**2**) in the case of the samples labeled as D (**a_1_**,**a_2_**), E (**b_1_**,**b_2_**), and F (**c_1_**,**c_2_**), recorded at the temperature of 273 °K (black symbol), 293 °K (red symbol), 323 °K (blue symbol), 333 °K (green symbol), 353 °K (magenta symbol), and 373 °K (orange symbol).

**Figure 12:**
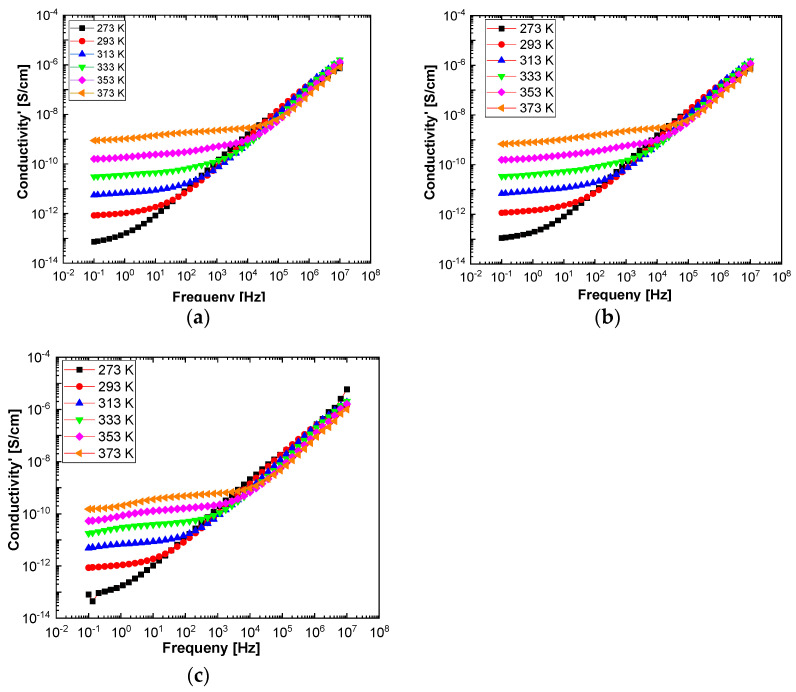
The electrical conductivity spectra of the samples labeled as A (**a**), B (**b**), and C (**c**) at the six temperatures: 273 °K (black symbol), 293 °K (red symbol), 323 °K (blue symbol), 333 °K (green symbol), 353 °K (magenta symbol), and 373 °K (orange symbol).

The ratios between the intensities of the Raman lines localized in the spectral ranges are as follows: (i) 1600–1650 and 2800–2950 cm^−1^ (I_1600–1650_/I_2800–2950_) is equal to ≈0.5; (ii) 1000–1200 and 2800–2950 cm^−1^ (I_1000–1200_/I_2800–2950_) is equal to ≈0.11; (iii) 1250–1350 and 2800–2950 cm^−1^ (I_1250–1350_/I_2800–2950_) is equal to ≈0.08; and (iv) 1400–1450 and 2800–2950 cm^−1^ (I_1400–1450_/I_2800–2950_) is equal to ≈0.13. According to Figure 3b–d, the following changes are induced to the TPU:TPO blend in the presence of CNs of the type BaSrTiO_3_: (i) a down-shift of the Raman line from 2920 cm^−1^ (the insert in Figure 3b) to 2908–2910 cm^−1^; (ii) an increase in the intensity of the Raman line situated in the spectral range 1600–1650 cm^−1^, the fact that induces a change of the I_1600–1650_/I_2800–2950_ ratio to ≈0.8; and (iii) the I_1000–1200_/I_2800–2950_, I_1250–1350_/I_2800–2950_, and I_1400–1450_/I_2800–2950_ ratios increase almost twice, as increasing the BaSrTiO_3_ concentration in the polymeric matrix from 6 wt % (0.28, 0.27, and 0.24, Figure 3b) to 12 wt % (0.23, 0.25, and 0.22, Figure 3c) and 25 wt % (0.25, 0.24, and 0.22, Figure 3d). These changes are similar to those reported in the case of the composites based on the TPU:TPO blend and BaTiO_3_ [12]. A careful analysis of Figure 3 highlights that the Raman line of BaSrTiO_3_ peaked at 525 cm^−1^ (Figure 3a), assigned to the A1(TO) vibrational mode [15], is shifted to 515–517 cm^−1^ in the case of the A, B, and C samples, i.e., when the CNs concentration as well as their crystallites size in the TPU:TPO thermoplastic polymers mass decreases. This result is in good agreement with our previous study reported on BaTiO_3_ [12], its origin being in the defects generated during the preparation of the A, B, and C samples, as above mentioned.

A careful analysis of Figure 4 highlights the following changes induced to the Raman spectrum of the TPU:TPO blend as a consequence of the increase of the SrTiO_3_ concentration in the mass of the D, E, and F samples: (i) an increase in the intensity of the Raman line localized in the spectral range 1600–1650 cm^−1^ inducing a variation of the I_1600–1650_/I_2800–2950_ ratio from ≈0.5 (the insert of Figure 3b) to 0.52 (Figure 4a), 1.24 (Figure 4b), and 1.46 (Figure 4c), when the CNs concentration is equal to 6 wt %, 12 wt %, and 25 wt %, respectively; (ii) the appearance of a new Raman line in the spectral range 1650–1700 cm^−1^, which often was attributed to vibrational modes of the C=O bonds in the COO− functional groups; (iii) an enhancement of the Raman line situated in the spectral range 1400–1500 cm^−1^ from 0.07 (the insert of Figure 3b) to 0.35 (Figure 4a), 0.8 (Figure 4b), and 0.7 (Figure 4c); and (iv) a gradual increase of the I_1000–1200_/I_2800–2950_, I_1250–1350_/I_2800–2950_, and I_1400–1450_/I_2800–2950_ ratios, when the SrTiO_3_ concentration is equal to 6 wt % (0.38, 0.32, and 0.35, Figure 4a), 12 wt % (0.84, 0.55, and 0.8, Figure 4b) and 25 wt % (0.71, 0.41, and 0.7, Figure 4c).

For a better explanation of above changes in Figure 5, we show the IR spectra of the composites based on the TPU:TPO blends and CNs of the type BaSrTiO_3_ and SrTiO_3_. According to the insert of Figure 5a, the main vibrational features of the TPU:TPO blend are shown in Table 2.

The ratios between the absorbances of the IR bands situated at: (i) 1016 and 1074 cm^−1^; (ii) 1221 and 1257 cm^−1^; (iii) 800 and 1074 cm^−1^ are equal to ≈0.69, ≈1.42, and ≈0.61, respectively. The increase in concentration of BaSrTiO_3_ in the composite weight containing the TPU:TPO blend induces the following changes in the IR spectrum of the thermoplastic polymers: (i) a gradual down-shift of the IR band from 800 to 791 cm^−1^, simultaneous with the increase in the absorbance, such that the ratio between the absorbance of the IR bands situated at 800 and 1074–1065 cm^−1^ is changed from 0.61 (insert of Figure 5a) to 0.65 (Figure 5a), 0.87 (Figure 5b), and 1.24 (Figure 5c); (ii) a progressive change of the complex IR band localized in the spectral range 980–1125 cm^−1^; the ratio between the absorbance of the two IR bands at 1014–1018 and 1065–1074 cm^−1^ varies from 0.69 (insert in Figure 5a) to 0.8 (Figure 5a), 1.1 (Figure 5b), and 1.34 (Figure 5c); and (iii) a decrease in the absorbance of the IR bands situated in the spectral range 1450–1750 cm^−1^, which is accompanied by the variation of the ratio between the absorbance of IR bands at 1221 and 1257 cm^−1^ from 1.42 (insert in Figure 5a) to 0.55 (Figure 5a), 0.43 (Figure 5b), and 0.21 (Figure 5c).

A careful analysis of Figure 5d highlights a similar behavior in the case of the A, B, and C samples, i.e., when the SrTiO_3_ concentration increases to 6 wt %, 12 wt %, and 25 wt % in the thermoplastic polymers matrix. These variations of vibrational properties of the TPU:TPO blend in the presence of the BaSrTiO_3_ and SrTiO_3_ CNs are found to be similar to those in the case of the TPU:TPO/BaTiO_3_ composites [12]. Taking into account all these, we conclude that both in the presence of the BaSrTiO_3_ and SrTiO_3_ CNs, the most reactive repeating units are those of TPU, the adsorption process of the thermoplastic polymer onto the CNs surface involving an exchange reaction similar to that reported in Ref. [12]. Moreover, this statement can be approved from XRD patterns by considering the decreasing ratio between the relative peak intensities of (110) planes of SrTiO_3_ and SrTiO_2.5_ from: (a) 3.32 (BaSrTiO_3_) to 1.81 (A sample) and (b) 11.38 (SrTiO_3_) to 2.3 (D sample). Additional information concerning the adsorption of the TPU:TPO thermoplastic polymers onto the CNs surface are shown in the following by anisotropic photoluminescence.

### 3.3. The Photoluminescence Properties of the Composites Based on TPU:TPO and the SrTiO_3_ and BaSrTiO_3_ CNs

Figure 6a,b show PL spectra of BaSrTiO_3_ and SrTiO_3_ CNs recorded at the excitation wavelength of 350 nm. Both BaSrTiO_3_ and SrTiO_3_ are characterized by an emission band with the maximum at 426 nm, having the intensity around 3.4 × 10^4^ and 1.98 × 10^4^ counts/s, respectively. The insert of the Figure 6c highlights that the emission band of the TPU:TPO blend is peaked at 465 nm having the intensity around of 3.01 × 10^7^ counts/s. According to our previous study, the position of the PL band of the TPU:TPO blend is dependent of the weight ratio of the two thermoplastic polymers [12]. Thus, when the TPU:TPO mass ratio is equal to 1:1 and 2:1, the PL band of the TPU:TPO blends is peaked at 486 nm and 465 nm, respectively. The maximum of these PL bands does not correspond to a sum effect of the PL bands of TPU and TPO, which have the maxima at 410 nm and 441 nm, respectively. This fact was explained as a consequence of the exchange reaction between the two thermoplastic polymers [12]. According to Figure 7a, the PL spectrum of the TPU:TPO blend shows three emission bands peaked at 3.02 eV (410 nm), 2.7 eV (459 nm), and 2.36 eV (525 nm), which originate in the electronic transitions belonging to the repeating units of TPU, TPO, and the TPU:TPO blend, respectively [12].

Returning to Figure 6, a down-shift of the PL band of the TPU:TPO blend from 465 nm (insert in Figure 6c) to ≈423 nm (Figure 6d) and ≈417 nm (Figure 6c) is reported to be induced in the presence of the BaSrTiO_3_ and SrTiO_3_, respectively. This behavior is similar to that reported in the case of the PL spectra of the TPU:TPO blends, when the TPU weight increases simultaneously with the decrease of the TPO weight in the TPU:TPO blend mass [12]. In the present case, an increase of the TPO weight in the TPU:TPO blend mass can be explained only if we accept that an interaction of the TPU:TPO blends with BaSrTiO_3_ CNs took place. A puzzling fact is that an emission band with the maximum at 438 nm was also reported in the case of the composites based on the TPU:TPO blend and BaTiO_3_ CNs 25 wt % [12]. For a better understanding of the PL band at ≈438 nm, Figure 7b shows a deconvolution of the PL spectrum of the B sample. In this last case, three emission bands peaked at 3.02 eV (≈410 nm), 2.82 eV (≈439 nm), and 2.7 eV (≈459 nm). The differences remarked in the case of the TPU:TPO blend and the B sample highlight clearly an up-shift of the emission bands associated to the electronic transitions belonging to the repeating units of TPO and the TPU:TPO blend. This result indicates that the BaSrTiO_3_ CNs interact with the repeating units of the type TPU and TPU:TPO. In this context, we note that the frequency separations between the PL bands, which peaked at (i) 3.02 and 2.82 eV and (ii) 2.82 and 2.7 eV, are equal to 1613 cm^−1^ (0.2 eV) and 968 cm^−1^ (0.12 eV), which are situated no longer to those peaked at 1614–1616 cm^−1^ and 966–974 cm^−1^ (Figure 3) belonging to the vibration modes of the aromatic structure stretching and out-of-plane benzene bending in TPU [24]. The higher intensity of the Raman line situated to 966–974 and 1614–1616 cm^−1^ suggests the presence of steric hindrance effects induced of the adsorption of thermoplastic polymers onto the CNs surface. An additional variation induced of the two CNs is the decrease in the intensity of the TPU:TPO blend PL band from 3.01 × 10^7^ counts/s (the insert in Figure 6c) to (i) 6.94 × 10^6^ counts/s (A sample, blue curve in Figure 6c), 5.93 × 10^6^ counts/s (B sample, red curve in Figure 6c), and 5.36 × 10^6^ counts/s (C sample, magenta curve in Figure 6c) and (ii) 8.32 × 10^6^ counts/s (D sample, blue curve in Figure 6d), 5.88 × 10^6^ counts/s (E sample, red curve in Figure 6c), and 4.56 × 10^6^ counts/s (F sample, magenta curve in Figure 6d). This behavior confirms the role of the BaSrTiO_3_ and SrTiO_3_ CNs as PL quenching agent of the TPU:TPO blend.

Figure 8 and Figure 9 show the PL spectra in polarized light of the A, B, C, D, E, and F samples. The black and red curves in Figure 8 and Figure 9 correspond to PL spectra recorded when the emission and excitation polarizors are both in horizontal (HH) and vertical (VV) position. According to the anisotropic PL measurements shown in Figure 8 and Figure 9, a dependence of PL spectra with the light polarization in the case of the A, B, C, D, E, and F samples is reported.

Considering the mathematic algoritm shown in Ref. [25], in the case of the single-phonon excitation, we calculate the anisotropy (r) and angle of the binding (θ_PL_) of the following: (i) the TPU:TPO thermoplastic polymers in the absence of the CNs to be equal to 0.302 and 23.7° [26]; (ii) the TPU:TPO thermoplastic polymers adsorbed onto BaSrTiO_3_ to be around of: (a) 0.08 and 46.9° for the A sample, (b) 0.03 and 51.5° for the B sample, and (c) 0.05 and 49.3° for the C sample; and (iii) the TPU:TPO thermoplastic polymers adsorbed onto SrTiO_3_ to be around of (a) 0.04 and 66° fo the D sample, (b) 0.61 and 52.4° for the E sample, and (c) 0.01 and 53.4° for the F sample. These results indicate that in the presence of CNs, an increase in θ_PL_ takes place, as a consequence of the exchange reaction between urethanic-type repeating units of the TPU:TPO blend and CNs. Depending on the type of CNs, BaSrTiO_3_, or SrTiO_3_, the biggest differences between θ_PL_ are reported for the composites having a concentration of CNs equal to 6 wt %. At higher CNs concentrations, i.e., 12 wt % and 25 wt %, the differences between θ_PL_ values are smaller as a consequence of the potential aggregation processes of nanoparticles during the preparation of the thermoplastic polymers/ceramic nanoparticles composites as free membranes.

### 3.4. The Dielectric and Conductive Properties of the Composites Based on TPU:TPO and the SrTiO_3_ and BaSrTiO_3_ CNs

The three main processes of dielectric relaxation (dipole, interfacial polarization, and electrode polarization) plus conduction mechanism can be observed and studied through the spectra of corresponding electrical quantities.

Figure 10 and Figure 11 show separately the spectra of dielectric constant and dielectric losses for the A, B, C, D, E, and F samples. From the entire range, six temperature values were uniform distributed as such: T = 273 °K, T = 293 °K, T = 323 °K, T = 333 °K, T = 353 °K, T = 373 °K. For the six samples, it is found that the spectra of dielectric constant, ε’(ω), and of the dielectric losses, ε’’(ω), shows common features. In the low-frequency region, a decreasing branch is observed, with increasing frequency, for both components of permittivity; the variation of the two quantities is of several orders of magnitude. A significant increase of the permittivity components at the mentioned frequency can be attributed to an electrical conduction mechanism through charge carriers, electrons, or ions. In the same spectra, in addition to electrical conduction, there is also a dielectric relaxation mechanism that manifests itself at higher frequencies, over 1 kHz.

It is best observed in the spectra of dielectric losses, in the form of a “hill” that has an ascending side, a maximum point, and a descending side. According to Figure 10 and Figure 11, one observes that the maximum point moves to higher frequencies as the temperature increases.

**Figure 13:**
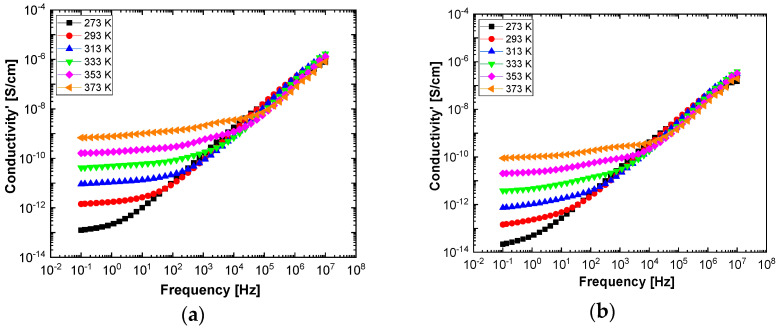

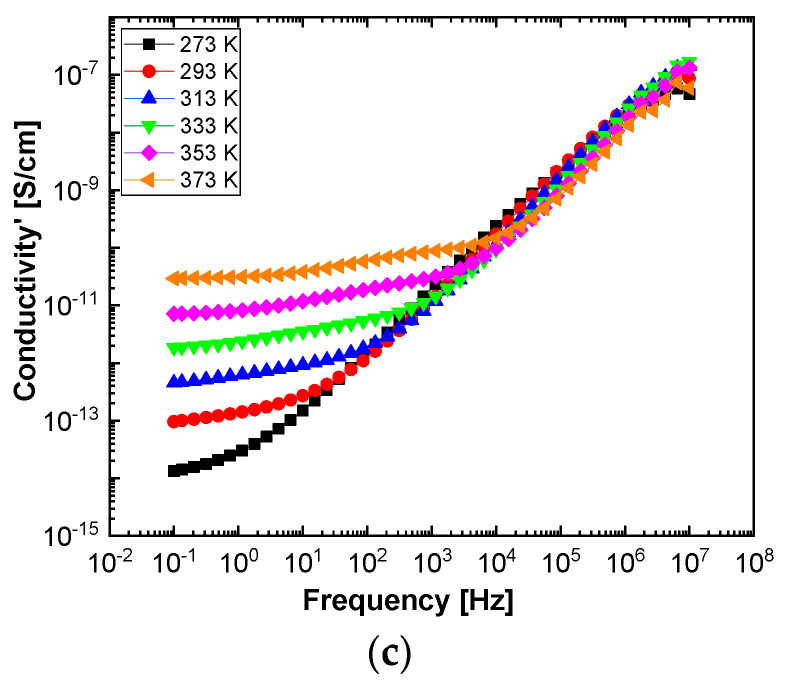
Spectra of electrical conductivity of the D (**a**), E (**b**), and F (**c**) samples recorded at the following six temperatures: 273 °K (black symbol), 293 °K (red symbol), 323 °K (blue symbol), 333 °K (green symbol), 353 °K (magenta symbol), and 373 °K (orange symbol).

In order to highlight properties related to electrical conductibility, we examine the shape of the electrical conductivity, σ’, as seen in Figure 12 and Figure 13. The same temperature values were chosen as in the case of permittivity. Regardless of the CNs type, i.e., BaSrTiO_3_ or SrTiO_3_, for all samples, i.e., A, B, C, D, E, and F, the conductivity spectra at different temperatures show an ascending branch at higher frequencies, over 100–1000 Hz, and a plateau region, where the conductivity has a small variation at lower frequencies, below 10–100 Hz. As the temperature increases, the plateau moves to the higher values of conductivity and expands more to higher frequencies. This is due to the electrical conductivity through free charge carriers and sustains the results shown in Figure 12 and Figure 13. Consequently, at low frequencies, the electrical conductivity has a constant value, while at high frequencies, it depends on the frequency, varying approximately as a power of it. It should be noted that similar spectra of electrical conductivity depending on temperature and frequency are found in a wide variety of homogeneous or heterogeneous samples in solid or liquid state (e.g., doped crystals that have ionic conduction, ionic glasses, polymers, amorphous semiconductors, solid state electrolytes, and so on). For the analysis of these spectra of macaroscopic conductivity, Joncher proposes the overlapping of two different contributions: a direct current (DC) conductivity, which is temperature dependent but is independent of frequency, and an alternating current component, which is frequency dependent but weakly temperature dependent. Thus, the real part of the electrical conductivity is written as the sum $\sigma \prime \left(\omega \right)=\sigma_{DC}+\sigma_{AC}\left(\omega \right)$, where the alternating current component is according to the law $\sigma_{AC}\left(\omega \right)=A\omega^{s}$, 0 < s < 1, which is called the “universal dielectric response” [27,28].

By processing spectra with certain model functions, which adequately describe the dielectric relaxation processes, we can obtain information about the different mechanisms of molecular dynamics. In our case, as permittivity and conductivity spectra are presented, the used fit function has the following mathematical expression [29,30,31,32]:

(1) $$\varepsilon *\left(\omega \right)=\varepsilon^{\prime }\left(\omega \right)-i\varepsilon^{\prime \prime }\left(\omega \right)=-i{\left(\frac{\sigma_{0}}{\varepsilon_{0}\omega }\right)}^{N}+\left[\frac{\Delta \varepsilon }{{\left(1+{\left(i\omega \tau_{c}\right)}^{\alpha }\right)}^{\beta }}+\varepsilon_{\infty }\right]$$

On the right side of the equation, the first term is given by the contribution of the electrical conductivity to the behavior of the samples, and in the right parenthesis is the Hvriliak–Negami fitting function. The fitting parameters have the following means: σ_0_ conductivity; N is a sub-unit exponent; ∆ε = ε_0_ − ε_∞_ is called dielectric strength, where

$$\varepsilon_{0}=\underset{\omega \to 0}{\lim}\;\varepsilon^{\prime }\left(\omega \right)$$

$$\varepsilon_{\infty }=\underset{\omega \to \infty }{\lim}\varepsilon^{\prime }\left(\omega \right)$$

τ_c_ = 1/ω_max_

The characteristic time is inversely proportional to the frequency (angular velocity) at which the dielectric losses have a maximum point (ε″(ω_max_) = ε″_max_); the sub-unit exponents, α, β called shape parameters, influence the extension (widening) and asymmetry of the relaxation curve around the dielectric losses maximum point; 0 < α ≤ 1, 0 < β ≤ 1. The parameter β is kept fixed during the fitting processes, with the value β = 1. The other parameters are free to be modified by the fitting program for optimization by the least-squares method. 

**Figure 14:**
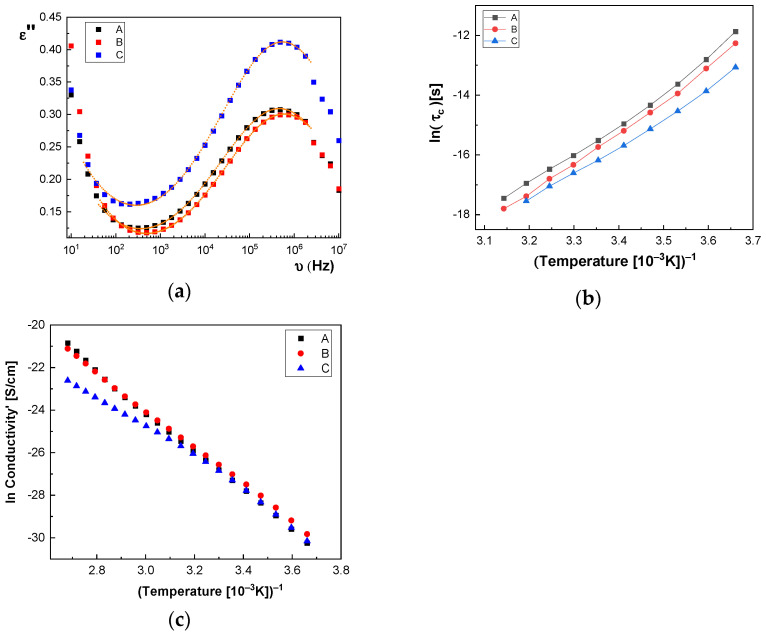
(**a**) Permittivity spectra of the A, B, and C samples at T = 293 °K; fitting functions—solid line; the experimental data—dotted curves.; (**b**) Arrhenius diagram: the dependence of relaxation time on the inverse of the temperature, ln(τ_c_) = f(1000/T); (**c**) Electrical conductivity Arrhenius plot.

**Figure 15:**
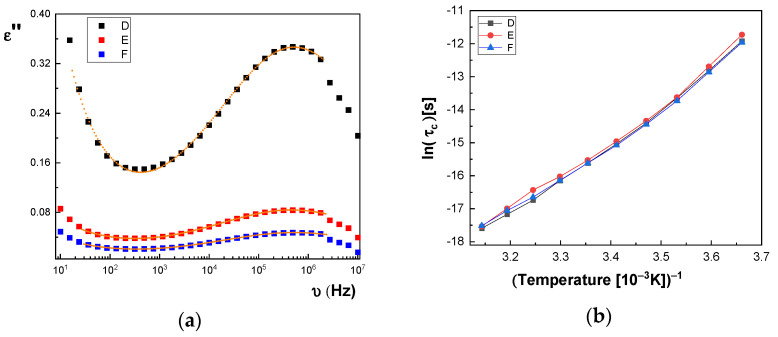

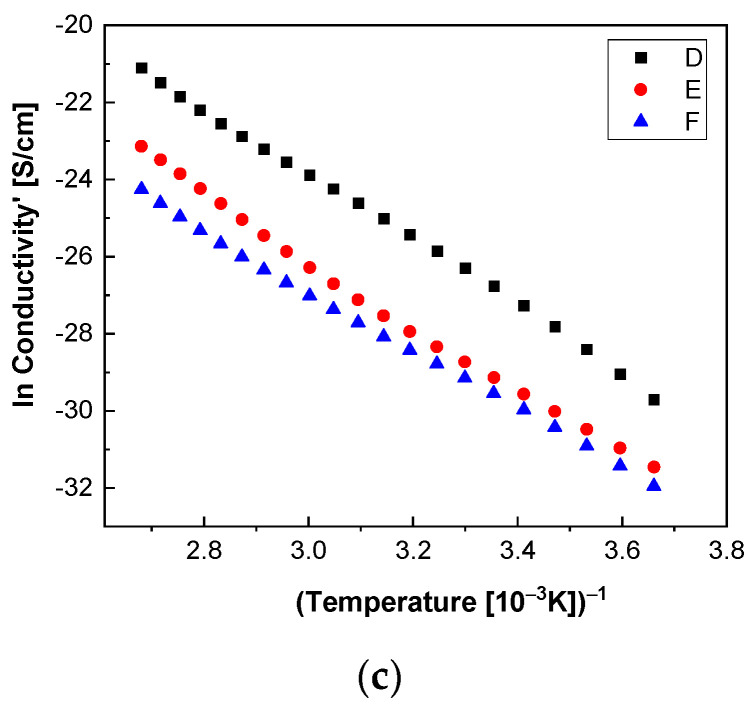
(**a**) Permittivity spectra of the D, E, and F samples at T = 293 °K; fitting functions—solid line; the experimental data—dotted curves.; (**b**) Arrhenius diagram: the dependence of relaxation time on the inverse of the temperature, ln(τ_c_) = f(1000/T); (**c**) Electrical conductivity Arrhenius plot.

In Figure 14a and Figure 15a, we represent on the same graph the spectra of dielectric losses for the A, B, C, D, E, and F samples over which are drawn the corresponding fitting functions. The temperature value is T = 293 °K. In this way, we can compare the relative position of the maximum points, as in Figure 14a and Figure 15a, but also the good overlap between the experimental data and fitting functions. The values obtained for characteristic time corresponding to maximum point to the left side of the graph: (a) the A sample, τ_c_ = 3.190 × 10^−7^ s; (b) the B sample, τ_c_ = 2.527 × 10^−7^ s; (c) the C sample, τ_c_ = 2.68 × 10^−7^ s; (d) the D sample, τ_c_ = 2.953 × 10^−7^ s; (e) the E sample, τ_c_ = 3.193 × 10^−7^ s; and (f) the F sample, τ_c_ = 2.831 × 10^−7^ s. For all samples, the shape parameter (α) has values between 0.284 and 0.48. It means that the spectrum dielectric losses shape around the maximum point is flattened (extended). The dielectric relaxation process is a non-Debye electrical dipole relaxation, in which the exponent has values different from 1. As a result of the fittings, we notice that the relaxation time depends on the temperature. The Arrhenius diagram contains the graphical representation of characteristic time as a function of inverse of the temperature: τ_c_ = f(1/T).

In the Arrhenius diagram, the fit function is given by Vogel–Fulcher–Tammann (VFT) empirical law [33,34,35,36]:

(2) $$\ln\left(\tau_{c}\right)=\ln\left(\tau_{\infty }\right)+\frac{W_{A}}{k_{B}\left(T-T_{V}\right)}$$

where the material constant, *W_A_*, is associated with the activation energy of dielectric or dipolar relaxation process, k_B_ is the Boltzmann constant, *T_V_* is the Vogel temperature, τ_∞_ is the characteristic time limit value when the temperature tends to very high values, $\tau_{\infty }=\underset{T\to \infty }{\lim}\left(\tau_{c}\right)$. The activation constant is equal to the slope of curve for higher temperature values (on left side of Figure 14b and Figure 15b), and the Vogel temperature produces a more pronounced variation at lower temperatures, which can be seen on the graph’s right side, (Figure 14b and Figure 15b). In a particular case where *T_V_* = 0, the above function obeys the Arrhenius law, $\ln\left(\tau_{c}\right)=\ln\left(\tau_{\infty }\right)+W_{A}/k_{B}T$, and the constant *W_A_* is equal to the activation energy. The VHT empirical law is generally used because it adapts much better to the data presented by a large number of different samples. As observed in Figure 14b, the characteristic time for the dielectric relaxation process has lower and lower values when passing from the A sample to the C sample, while in Figure 15b, the curves corresponding to the D, E, and F samples are very close. Based on the values of fitting parameters, in Table 3 and Table 4, we can make a comparison between the samples, which differ from each other by CNs concentration. First, we note that τ_∞_ is very sensitive to the fitting process; for this reason, it can have important fluctuations, and we cannot take it into account to compare the samples. The activation constant, *W_A_*, globally highlights the characteristic time dependence on temperature and, as a result, it is less sensitive to the fitting performed.

According to Table 3, one observes that there is a tendency for the W_A_ value to increase with increasing concentration of BaSrTiO_3_ nanoparticles in the case of the A, B, and C samples. A different behavior, the slight decrease of the *W_A_* value, is noted in the case of the D, E, and F samples containing the SrTiO_3_ nanoparticles (see Table 4). Moreover, there is a tendency to decrease the Vogel temperature value, *T_V_*, for samples A, B, and C, depending on the concentration, and a small trend to increase the *T_V_* for samples D, E, and F, respectively. The high obtained value of *T_V_* (100 °K–200 °K) is specific to the samples that present “the glass transition”, as is the case of the macromolecular compounds [37,38]. Returning to Figure 12 and Figure 13, the conductivity spectra show that it depends on the temperature. The DC electric conductivity is based on a mechanism that is thermic activated, governed by the Arrhenius law for a large amount of samples. From Arrhenius plot, in which the conductivity is represented as a function of the inverse of the temperature, we can obtain the activation energy of the electrical conduction mechanism, i.e., the necessary energy to generate electrical charge carriers. The conductivity fitting function is defined in accordance with Arrhenius law [39,40], as follows:

(3) $$\ln\left(\sigma_{DC}\right)=\ln\left(\sigma_{\infty }\right)-\frac{E_{a}}{k_{B}T}.$$

By fitting the curves with the Arrhenius function, as shown in Equation (3), the activation energy of the electrical conductivity (E_a_) has the value for: (i) A sample, 80.277 × 10^−3^ eV; (ii) B sample, 74.806 × 10^−3^ eV; (iii) C sample, 74.637 × 10^−3^ eV; (iv) D sample, 72.96 × 10^−3^ eV; (v) E sample, 72.944 × 10^−3^ eV; and (vi) F sample, 67.409 × 10^−3^ eV. Figure 14c and Figure 15c highlight a straight line for the A and B samples as well as in the case of the D, E, and F samples, which indicates an Arrhenius-type activation. The graph of the C sample (Figure 14c) seems to have a more pronounced curvature, which suggest the existence of two possible conduction activation mechanisms, i.e., one at lower temperatures and another at higher temperatures. The conductivity values of the A, B, and C samples are not significantly different (see Figure 14c). All conductivities curves overlap at lower temperature. Therefore, the change of the BaSrTiO_3_ concentration in the sample does not contribute to the mechanism of generating electric charge carriers. For D, E, and F samples, the conductivity shows a decreasing tendency with the SrTiO_3_ concentration of several orders of magnitude from one sample to another. 

Summarizing these results, we can conclude that the A, B, C, D, E, and F samples show two mechanisms: (a) one regarding the type of electrical conduction, which is manifested by the simultaneous increase of the dielectric constant and dielectric losses with several orders of magnitude at low frequencies as well as the appearance of a plateau region in the of electrical conductivity in same frequency range; a variation of the electrical conductivity is observed only for the D, E, and F samples, without a change in the charge carriers’ generation mechanism; and (b) another one regarding the type of electric dipolar relaxation process that appears at high frequencies, which is best observed in dielectric losses spectra by the presence of a maximum point; it is a non-Debye relaxation mechanism; dipole-relaxation activation energy shows an increase with increasing CNs concentration only for the A, B, and C samples.

## 4. Conclusions

In this work, we have reported new results concerning the structural, optical, dielectric, and conductive properties of the composites based on the TPU:TPO thermoplastic polymers and CNs of the type BaSrTiO_3_ or SrTiO_3_. The following conclusions can be highlighted according to the above results: (i) a cubic crystalline structure of the BaSrTiO_3_ or SrTiO_3_ CNs and an amorphous phase in the case of the TPU:TPO blends was highlighted by X-ray diffraction; (ii) the changes reported by Raman scattering and IR spectroscopy, to be induced to the TPU:TPO blends in the presence BaSrTiO_3_ and SrTiO_3_, respectively, have indicated an exchange reaction at the interface of the urethane-based repeating units of the thermoplastic polymers with CNs; (iii) according to PL studies, CNs play the role of the TPU:TPO blends PL quenching agent; (iv) the anisotropic PL studies have highlighted significant changes in the angle of the binding of the thermoplastic polymers onto the CNs surface, regardless of the filler concentration; (v) dielectric spectroscopy studies have indicated in the case of the composites based on the TPU:TPO blends and CNs of the type of BaSrTiO_3_ and SrTiO_3_ that they have two molecular mechanisms, i.e., one is of the type of the electrical conduction and another is specific to the dielectric–dipolar relaxation processes. The main application of these composite materials envisaged to be developed is in the field of the 3D printed energy harvesting devices.

## Figures and Tables

**Table 1:** The vibrational modes active in the Raman spectrum of the TPU:TPO blend.

**Wavenumbers (ν, cm^−1^)**	**Assignment of Vibrational Modes [16,17,18]**
1301	deformation CH-urethane amide in TPU
1436	symmetrical stretching of N = C=O + deformation CH_2_ in TPU
1616	aromatic structure stretching in TPU
2867–2912	C-H stretching in TPO

**Wavenumbers (ν, cm^−1^)**

**Assignment of Vibrational Modes [16,17,18]**

**Table 2:** The vibrational modes active in the IR spectrum of the TPU:TPO blend.

Wavenumbers (ν, cm^−1^)	Assignment of Vibrational Modes [19,20,21,22,23,24]
800	N-H bending
1016	–CH_3_ rocking in TPO
1074	C(O)−OC stretching in TPU
1221	ether group stretching in TPU
1257	CH bending in TPO
1530	stretching benzene ring
1701	H-bonded urethane C=O groups

**Table 3:** Fit parameters in the VFH function for the A, B, and C samples.

Fit Parameters in the VFH Function	A Sample	B Sample	C Sample
$\tau_{\infty }$ [s]	3.878 × 10^−13^	4.321 × 10^−13^	2.30 × 10^−13^
$W_{A}$ [eV]	1.306 × 10^−1^	1.487 × 10^−1^	1.456 × 10^−1^
$T_{V}$ [K]	1.823 × 10^2^	1.675 × 10^2^	1.726 × 10^2^
MSD [%]	2.02	2.22	1.55

**Table 4:** Fitting parameters that appear in the VFH function.

Fit Parameters in the VFH Function	D Sample	E Sample	F Sample
$\tau_{\infty }$ [s]	4.242 × 10^−13^	6.328 × 10^−13^	2.11 × 10^−12^
$W_{A}$ [eV]	1.235 × 10^−1^	1.19 × 10^−1^	9.928 × 10^−2^
$T_{V}$ [K]	1.868 × 10^2^	1.885 × 10^2^	1.958 × 10^2^
MSD [%]	1.01	2.106	0.85

## Data Availability

Samples are available from authors.
